# Case report: Rapid onset, ischemic-type gastritis after initiating oral iron supplementation

**DOI:** 10.3389/fmed.2022.1010897

**Published:** 2022-11-03

**Authors:** Regina M. Koch, Stefan Tchernodrinski, Daniel R. Principe

**Affiliations:** ^1^Department of Medicine, University of Illinois College of Medicine, Chicago, IL, United States; ^2^Medical Scientist Training Program, University of Illinois College of Medicine, Chicago, IL, United States

**Keywords:** gastritis, oral iron supplementation, gastroesophageal reflux disease, emergency medicine, medication adverse effect, gastrointestinal tract

## Abstract

Oral iron supplements are commonly administered to patients with chronic iron deficiency anemia. This approach is generally well-tolerated, causing only mild adverse effects. Rarely, oral iron supplementation can cause more severe symptoms, one of the most concerning being acute gastritis. This predominantly affects elderly patients and is extremely uncommon in young, otherwise healthy people. Here, we report the case of a 43-year-old woman who presented with upper gastrointestinal (GI) symptoms and iron deficiency anemia and was started on oral iron supplementation following the resolution of her acute symptoms. She soon re-presented with a severe, *Helicobacter pylori*-negative gastritis with iron deposition on histology. These new onset symptoms resolved rapidly with cessation of iron supplements, consistent with iron pill gastritis. In addition to the limited body of literature describing iron pill gastritis, this case serves as a reminder that any patient receiving oral iron supplementation is at a potential risk for gastritis, particularly in the setting of an ongoing GI pathology. Hence, it is important to provide continued follow-up for patients receiving iron supplementation regardless of age or comorbidity, particularly in the weeks following the start of the treatment.

## Introduction

Oral iron supplements are widely used in the treatment of patients with iron deficiency anemia (1). Iron supplements are generally well-tolerated, with many patients experiencing only minor side effects including nausea/vomiting, metallic taste, staining of the teeth, or gastrointestinal (GI) distress (2). However, in rare instances, oral iron supplementation can lead to more severe symptoms, namely, acute gastritis. This is commonly referred to as “iron pill gastritis” and is diagnosed in a patient receiving iron supplementation who develops erosive gastritis with iron deposition on histopathology (3, 4). Although iron-induced mucosal injury is rare in all patients, it is most common in the elderly (5), with only a few reported instances in young patients. Here, we report the case of a 43-year-old woman with iron deficiency anemia and unmanaged gastroesophageal reflux disease (GERD) who presented with melena and coffee-ground emesis. After her acute symptoms were resolved, she was administered oral iron supplementation with plans to follow-up in the outpatient setting. However, she rapidly developed upper GI bleeding and a severe, *Helicobacter pylori* (*H. pylori*)-negative gastritis with iron deposits on histology. This case serves as an important reminder that, though rare, any patient receiving oral iron supplementation is at a risk for gastritis. Accordingly, it is essential to provide continued follow-up for patients receiving iron supplementation independent of age or comorbidity.

## Case presentation

A 43-year-old woman presented to the emergency department complaining of intermittent melena, and coffee-ground emesis for the past two weeks. She also reported an unintentional 25-pound weight loss in recent months, which she attributed to nausea and decreased appetite. She had been diagnosed with severe GERD approximately 18 months earlier, though she was not taking any medication. Physical examination was unremarkable, and negative for abdominal pain or peritoneal signs. Lab work was significant only for normocytic anemia with hemoglobin of 10.1 g/dl (normal range 12–15.5 g/dl) with a mean corpuscular volume of 97 fl (normal range 80–100 fl). Fetal occult blood testing was negative, LDH was within normal limits, and blood urea nitrogen to creatinine ratio was unremarkable at 14:1. Additional workup showed a low serum iron of 31 mcg/dL (normal range 60–170 mcg/dL) with low transferrin (123 mg/L, normal range 11 to 307 mg/L), low total iron binding capacity (172 mcg/dL, normal range 240–450 mcg/dL), low transferrin saturation (18%, normal range 20–50%), and a ferritin of 87 ng/mL (normal range 5–116 ng/mL). After her acute symptoms had resolved, the patient was started on oral ferrous sulfate of 325 mg BID and scheduled for a non-emergent endoscopy given the likelihood of a potential GI bleed.

**FIGURE 1:**
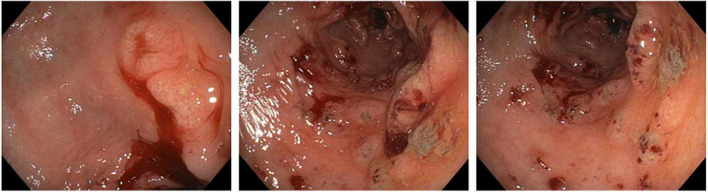
Esophagogastroduodenoscopy showing atrophic gastritis with nodular mucosa and multiple non-bleeding ulcerations.

**FIGURE 2:**
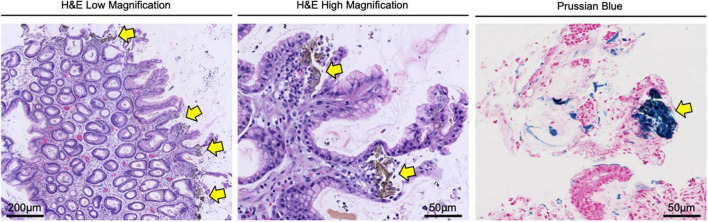
Gastric biopsy confirming the diagnosis of iron pill gastritis. Gastric lesions were biopsied and stained with H&E, showing inflammation with pits of brown pigment (yellow arrows) consistent with iron gastropathy. Tissues were also stained *via* Prussian blue, confirming the presence of iron crystals (yellow arrows) in the gastric mucosa.

After five days, the patient reported bright red blood in her stool. On re-evaluation, her hemoglobin was now 7.5 g/dl, and physical examination was significant for upper quadrant and left flank tenderness. Given concern for a new or worsening upper GI bleed, an urgent esophagogastroduodenoscopy (EGD) was performed which revealed atrophic gastritis with nodular and thickened mucosa, and multiple non-bleeding ulcerations in the gastric body, antrum, and prepyloric regions (Figure 1). A hemostatic clip was placed in the second portion of the duodenum, and ulcerations were biopsied, showing inflammation with pits of brown pigment consistent with iron gastropathy (Figure 2). Biopsies showed no evidence of intestinal metaplasia or lymphoma, stained negative for *H. pylori*, and positive for iron crystals *via* Prussian blue staining (Figure 2). Based on these observations and recent criteria regarding histologic subtypes for drug-induced GI lesions (6), the patient was diagnosed with an ischemic-type iron pill gastritis and oral ferrous sulfate was discontinued immediately. The patient was treated conservatively with omeprazole (40 mg PO daily) for her GERD, and experienced the resolution of her symptoms over the next two days, reporting no further nausea, vomiting, hematemesis, melena, or hematochezia.

## Discussion

Iron-induced mucosal injuries to the upper GI tract are well-documented. These events involve GI necrosis and strictures following an iron overdose and generally occur due to comorbidities that cause the excessive accumulation of iron including hemochromatosis, cirrhosis, and multiple blood transfusions (4, 5). However, for patients receiving standard dose iron supplements without systemic iron overload, the etiology of mucosal injury is poorly understood.

To this end, a seminal study explored the effects of standard-dose oral iron therapy on the upper GI tract using 27 healthy volunteers. In this study, 14 participants underwent baseline endoscopy with a biopsy of the stomach and duodenum and provided a stool sample. They were then administered standard dose oral iron supplementation for two weeks, followed by a repeat endoscopy/biopsy and stool sample collection. Thirteen additional participants provided a pre-treatment stool sample, provided a pretreatment stool sample and were administered standard dose oral iron supplementation for one week, and a posttreatment stool sample was collected. All participants developed dark stools posttreatment, and nausea and diarrhea were ubiquitous. One patient had a potentially trace-positive hemoccult test. However, the authors noted that several participants showed changes in posttreatment endoscopy of the stomach, mostly in the form of erythema, small areas of subepithelial hemorrhage, and (in two patients) gastric erosions (7).

A subsequent study of 1,300 upper GI tract biopsies from 33 patients identified crystalline iron deposition in 12/1,300 biopsies, and mucosal damage in 9/1,300. The authors noted that the areas of mucosal ulceration had a significant overlap with those of iron deposition, particularly for patients with associated upper GI disorders. The authors therefore concluded that therapeutic iron supplementation can both induce and exacerbate erosive mucosal injury (8). Despite these and other studies, it remains unclear how iron pill gastritis occurs or why certain patients are affected. Recent evidence suggests that this involves iron oxidation from ferrous to ferric form, leading to epithelial injury of the esophagus and stomach (9), which can lead to mucosal injury similar to that caused by chemical burns (10). However, this area warrants continued exploration, particularly as the field reaches a consensus that iron pill gastritis is likely underdiagnosed (3, 4).

Importantly, as our patient presented with acute GI symptoms, our case raises the question as to whether oral iron supplementation led to a new ulcerative gastritis, or exacerbated an existing, more insidious gastritis. In addition, it also raises questions as to whether the standard approach to iron supplementation is appropriate for all patients. For years, the treatment guidelines for iron deficiency anemia in adults have recommended a daily intake of 100–200 mg of oral iron, administered as either one or two doses. However, emerging evidence suggests that less frequent dosing may be more effective than daily dosing. For example, two recent open-label trials measured iron absorption in iron-deficient, non-anemic adult women receiving various dosing regimens of oral iron. The authors found that total iron absorption was superior in women receiving every-other-day iron dosing when compared to those receiving daily dosing. Additionally, there was no significant difference in iron absorption between women receiving a single daily dose vs. twice-daily divided doses (11).

A subsequent study in 19 women with iron deficiency anemia also determined that every-other-day iron dosing improved overall iron absorption when compared to once-daily dosing. The authors also reported a 40% decrease in Grade I and II GI side effects (abdominal pain, nausea, vomiting, and diarrhea), although this difference was not statistically significant likely due to the small sample size (12). Hence, this too warrants continued investigation, particularly regarding treatment-related adverse events in vulnerable patient populations.

Finally, there are also emerging data suggesting that intravenous (IV) iron sucrose may have a better safety profile and efficacy than oral iron supplements (13). For example, oral iron supplements are associated with higher GI side effects than either IV iron or placebo (14, 15). Classically, this approach has been reserved for patients with chronic iron deficiency anemia and comorbid chronic kidney disease, irritable bowel disease, or those who develop intolerable GI side effects from oral iron supplementation (16–18). However, given the favorable toxicity profile, IV administration may provide a safer and reasonable alternative to oral iron supplementation in complex patients like ours. While this requires further study, this case serves as an important reminder that careful follow-up is required for all patients started on iron supplementation, particularly those with ongoing upper GI pathologies.

## Data availability statement

The original contributions presented in this study are included in the article/supplementary material, further inquiries can be directed to the corresponding author.

## Ethics statement

Written informed consent was obtained from the individual(s) for the publication of any potentially identifiable images or data included in this article.

## Author contributions

RK and DP drafted the manuscript. ST edited the manuscript. All authors read and approved the final version of the manuscript.

## References

[1] Zimmermann MB Hurrell RF. Nutritional iron deficiency. *Lancet.* (2007) 370:511–20. 10.1016/S0140-6736(07)61235-5 17693180

[2] Nguyen M Tadi P. *Iron Supplementation.* Treasure Island, FL: StatPearls (2022). 32491308

[3] Onorati M Nicola M Renda A Lancia M Di Nuovo F. Iron overload in gastric mucosa: underdiagnosed condition rarely documented in clinical and pathology reports. *Cureus.* (2020) 12:e8234. 10.7759/cureus.8234 32601552 PMC7316405

[4] Sunkara T Caughey ME Nigar S Olivo R Gaduputi V. Iron pill gastritis: an under diagnosed condition with potentially serious outcomes. *Gastroenterol Res.* (2017) 10:138–40. 10.14740/gr804w 28496538 PMC5412550

[5] Haig A Driman DK. Iron-induced mucosal injury to the upper gastrointestinal tract. *Histopathology.* (2006) 48:808–12. 10.1111/j.1365-2559.2006.02448.x 16722929

[6] De Petris G Caldero SG Chen L Xiao SY Dhungel BM Spizcka AJ Histopathological changes in the gastrointestinal tract due to medications: an update for the surgical pathologist (part II of II). *Int J Surg Pathol.* (2014) 22:202–11. 24021900 10.1177/1066896913502230

[7] Laine LA Bentley E Chandrasoma P. Effect of oral iron therapy on the upper gastrointestinal tract. A prospective evaluation. *Dig Dis Sci.* (1988) 33:172–7. 10.1007/BF01535729 3257437

[8] Abraham SC Yardley JH Wu TT. Erosive injury to the upper gastrointestinal tract in patients receiving iron medication: an underrecognized entity. *Am J Surg Pathol.* (1999) 23:1241–7. 10.1097/00000478-199910000-00009 10524525

[9] Aruoma OI Halliwell B. Superoxide-dependent and ascorbate-dependent formation of hydroxyl radicals from hydrogen peroxide in the presence of iron. Are lactoferrin and transferrin promoters of hydroxyl-radical generation? *Biochem J.* (1987) 241:273–8. 10.1042/bj2410273 3032157 PMC1147552

[10] Hashash JG Proksell S Kuan SF Behari J. Iron pill-induced gastritis. *ACG Case Rep J.* (2013) 1:13–5. 10.14309/crj.2013.7 26157809 PMC4435261

[11] Stoffel NU Cercamondi CI Brittenham G Zeder C Geurts-Moespot AJ Swinkels DW Iron absorption from oral iron supplements given on consecutive versus alternate days and as single morning doses versus twice-daily split dosing in iron-depleted women: two open-label, randomised controlled trials. *Lancet Haematol.* (2017) 4:e524–33. 10.1016/S2352-3026(17)30182-5 29032957

[12] Stoffel NU Zeder C Brittenham GM Moretti D Zimmermann MB. Iron absorption from supplements is greater with alternate day than with consecutive day dosing in iron-deficient anemic women. *Haematologica.* (2020) 105:1232–9. 10.3324/haematol.2019.220830 31413088 PMC7193469

[13] Das SN Devi A Mohanta BB Choudhury A Swain A Thatoi PK. Oral versus intravenous iron therapy in iron deficiency anemia: an observational study. *J Fam Med Prim Care.* (2020) 9:3619–22. 10.4103/jfmpc.jfmpc_559_20 PMC7567229 33102339

[14] Cancelo-Hidalgo MJ Castelo-Branco C Palacios S Haya-Palazuelos J Ciria-Recasens M Manasanch J Tolerability of different oral iron supplements: a systematic review. *Curr Med Res Opin.* (2013) 29:291–303. 10.1185/03007995.2012.761599 23252877

[15] Tolkien Z Stecher L Mander AP Pereira DI Powell JJ. Ferrous sulfate supplementation causes significant gastrointestinal side-effects in adults: a systematic review and meta-analysis. *PLoS One.* (2015) 10:e0117383. 10.1371/journal.pone.0117383 25700159 PMC4336293

[16] Cooke M Lamplugh A Naudeer S Edey M Bhandari S. Efficacy and tolerability of accelerated-dose low-molecular-weight iron dextran (cosmofer) in patients with chronic kidney disease. *Am J Nephrol.* (2012) 35:69–74. 10.1159/000334877 22189072

[17] Macdougall IC. Strategies for iron supplementation: oral versus intravenous. *Kidney Int Suppl.* (1999) 69:S61–6. 10.1046/j.1523-1755.1999.055Suppl.69061.x 10084288

[18] Auerbach M Ballard H Glaspy J. Clinical update: intravenous iron for anaemia. *Lancet.* (2007) 369:1502–4. 10.1016/S0140-6736(07)60689-8 17482969

